# Toxic Effects of Mycotoxin Fumonisin B1 at Six Different Doses on Female BALB/c Mice

**DOI:** 10.3390/toxins14010021

**Published:** 2021-12-29

**Authors:** Zhiwei Chen, Fan Zhang, Lin Jiang, Zihan Chen, Hua Sun

**Affiliations:** State Key Laboratory of Bioactive Substance and Function of Natural Medicines, Institute of Materia Medica, Chinese Academy of Medical Sciences & Peking Union Medical College, 1# Xiannongtan Street, Xicheng District, Beijing 100050, China; chenzw@imm.ac.cn (Z.C.); AFUN008034@163.com (F.Z.); gillianjiang@imm.ac.cn (L.J.); chenzihan@imm.ac.cn (Z.C.)

**Keywords:** fumonisin B1, BALB/c mice, hepatotoxicity, nephrotoxicity, haematological toxicity, regulatory limit

## Abstract

Background: Fumonisin B1 (FB1) is one of the most common mycotoxins contaminating feed and food. Although regulatory limits about fumonisins have been established in some countries, it is still very important to conduct research on lower doses of FB1 to determine the tolerance limits. The aim of this study was to investigate the effects of different concentrations of FB1, provide further evidence about the toxic doses- and exposure time-associated influence of FB1 on mice, especially low levels of FB1 for long-term exposure. Methods: Female BALB/c mice were treated intragastrically (i.g.) with fumonisin B1 (FB1) solutions (0 mg/kg body weight (BW), 0.018 mg/kg BW, 0.054 mg/kg BW, 0.162 mg/kg BW, 0.486 mg/kg BW, 1.458 mg/kg BW and 4.374 mg/kg BW) once a day for 8 weeks to obtain dose- and time-dependent effects on body and organ weights, hematology, blood chemical parameters and liver and kidney histopathology. Results: After the long-term administration of FB1, the body weights of the mice tended to decrease. Over time, FB1 first increased the relative spleen weight, then increased the relative kidney weight, and finally increased the relative liver weight. The mean corpuscular volume (MCV), mean corpuscular hemoglobin (MCH), hemoglobin (HGB), white blood cells (WBC), platelets (PLT), and mean platelet volume (MPV) were significantly elevated after treatment with FB1 for 8 weeks. Moreover, exposure time-dependent responses were found for aspartate aminotransferase (AST), alanine aminotransferase (ALT) and alkaline phosphatase (ALP) level, which were coupled with hepatic histopathological findings, necroinflammation and vacuolar degeneration and detrital necrosis. Linear dose response was also found for liver histopathology, in which, even the minimum dose of FB1 exposure also caused changes. Renal alterations were moderate compared to hepatic alterations. Conclusion: In conclusion, we demonstrated the systemic toxic effects of different doses of FB1 in female BALB/c mice at different times. Our data indicated that the effects observed in this study at the lowest dose tested are discussed in relation to the currently established provisional maximum tolerable daily intake (PMTDI) for fumonisins. This study suggested that recommendations for the concentration of FB1 in animals and humans are not sufficiently protective and that regulatory doses should be modified to better protect animal and human health. The toxicity of FB1 needs more attention.

## 1. Introduction

Mycotoxins, the secondary metabolites mainly produced by *Aspergillus*, *Penicillium*, and *Fusarium*, are highly poisonous substances in animals and humans. They are capable of causing mycotoxicosis [1], involving acute toxic, carcinogenic, mutagenic, teratogenic, immunotoxic, and estrogenic effects [2]. Since the discovery of the aflatoxins in the 1960s, an increasing number of mycotoxins have been characterized, including deoxynivalenol, T2 toxin, fumonisins, ochratoxin, and zearalenone [3].

Fumonisins (FBs) are a group of hydrophilic mycotoxins produced by *Fusarium verticillioides* and its related species that commonly contaminate corn, sorghum, related grains and even the traditional Chinese medicines (TCMs) throughout the world [4]. Until now, the fumonisins characterized since 1988 can be divided into four major groups as fumonisin A, B, C and P series [5]. Among these four groups, the most abundant and toxic fumonisin analog is fumonisin B1 (FB1), which contributes to approximately 70% of FBs and is one of the most common mycotoxins contaminating feed and food [6]. FB1 has been classified by the International Agency for Research on Cancer (IARC) as a Group 2B possibly carcinogenic to humans [2]. Regulatory limits on fumonisins have been established in some countries. In the European Union, the maximum level of total FBs (FB1 + FB2) range from 200 μg/kg in processed maize-based foods to 2000 μg/kg in unprocessed corn products [7]. The Food and Agriculture Organization/World Health Organization (FAO/WHO) specified a tolerable daily intake (TDI) of 2 μg/kg BW/day for fumonisins (FB1, FB2 and FB3, alone or by combination) [8].

More evidence indicates that FB1 is neurotoxic [9], nephrotoxic, hepatotoxic [6], hepatocarcinogenic [10] and immunotoxic [11]. As a potential hazardous contaminant, FB1 has been shown to cause the production of equine leukoencephalomalacia (ELEM) and porcine pulmonary edema (PPE) [4,12]. The association of the intake of fumonisins with human neural tube defects (NTDs) in the fetus has also been shown in regions where maize is consumed as a major food source [13]. It is also regarded as a high incidence of human esophageal cancer [12]. The contamination of food [14,15], feed [16,17] and traditional Chinese medicines (TCMs) [18] with fumonisins has been an increasingly serious concern in our society.

The aim of this study was to investigate the effects of different concentrations of FB1, including a concentration corresponding to the PMTDI (provisional maximum tolerable daily intake) of 2 µg/kg BW for FB1, FB2 and FB3, alone or in combination by the Joint FAO/WHO Expert Committee on Food Additives (JECFA) [8] on food. These six different concentrations of FB1 solutions (0.018 mg/kg BW, 0.054 mg/kg BW, 0.162 mg/kg BW, 0.486 mg/kg BW, 1.458 mg/kg BW and 4.374 mg/kg BW) were continuously administered to female BALB/c mice, which are more sensitive to the toxicity of FB1 [19,20].

## 2. Results

### 2.1. Effects of FB1 on the General Health and Body and Organ Weights of Mice

**Figure 1:**
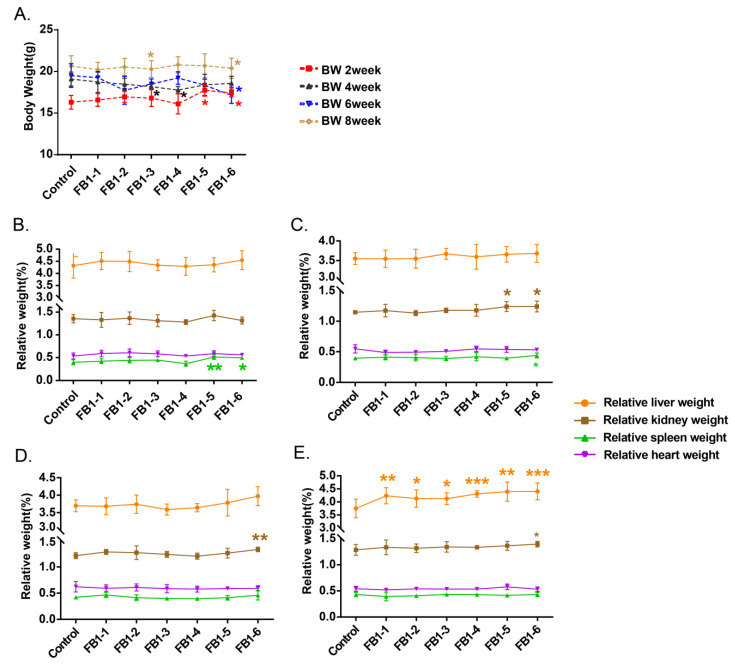
Effects of FB1 on body and organ weights of mice. (**A**) Mean body weight of mice exposed to different levels of FB1 for 2, 4, 6, and 8 consecutive weeks. (**B**–**E**) Relative organ weights of mice exposed to different levels of FB1 for 2, 4, 6 and 8 weeks. Values are shown as the mean ± SEM; 2 weeks (*n* = 5), 4 weeks (*n* = 6–7), 6 weeks (*n* = 5), and 8 weeks (*n* = 9). FB1-1 indicates 0.018 mg/kg BW, FB1-2 indicates 0.054 mg/kg BW, FB1-3 indicates 0.162 mg/kg BW, FB1-4 indicates 0.486 mg/kg BW, FB1-5 indicates 1.458 mg/kg BW and FB1-6 indicates groups 4.374 mg/kg BW; * indicates *p* < 0.05, ** indicates *p* < 0.01, *** indicates *p* < 0.001, vs. the control group.

All mice were weighed once two weeks before being sacrificed. All of the body weights (BWs) of the mice are shown in Figure 1A. When mice were fed with FB1 for 2 weeks, body weights of FB1-5 (1.458 mg/kg BW) and FB1-6 (4.374 mg/kg BW) were higher than the control group, whereas after 4 weeks exposure, FB1-3 and FB1-4 body weight resulted notably lower when compared to the control. After 6 and 8 weeks of FB1 treatment, the body weights of mice in the FB1-6 (4.374 mg/kg BW) group were significantly lower than those in the control group.

As regards the organ weights, only spleen of FB1-5 (1.458 mg/kg BW) and FB1-6 (4.374 mg/kg BW) showed a significant increase in its weight after 2 weeks’ exposure (Figure 1B). After 4 weeks, in addition to the relative spleen weights, the relative kidney weights of mice also presented an obvious increase in the high dose of FB1 (FB1-5 (1.458 mg/kg BW) and FB1-6 (4.374 mg/kg BW)). For mice treated with FB1 for 6 weeks, only the relative kidney weights in the FB1-6 group showed a significant increase compared with the control group. For mice treated with FB1 for 8 weeks, the relative kidney weights in the FB1-6 (4.374 mg/kg BW) group still showed a significant increase compared with the control group (Figure 1C). In addition, there was an obvious change in relative liver weight. The mice in all FB1-treated groups showed significantly higher relative liver weights than the control group (Figure 1D). From beginning to end, the relative heart weights of mice in each FB1-treated group showed no obvious changes compared to the control group (Figure 1E).

### 2.2. Effects of FB1 on Hematology of Mice

**Figure 2:**
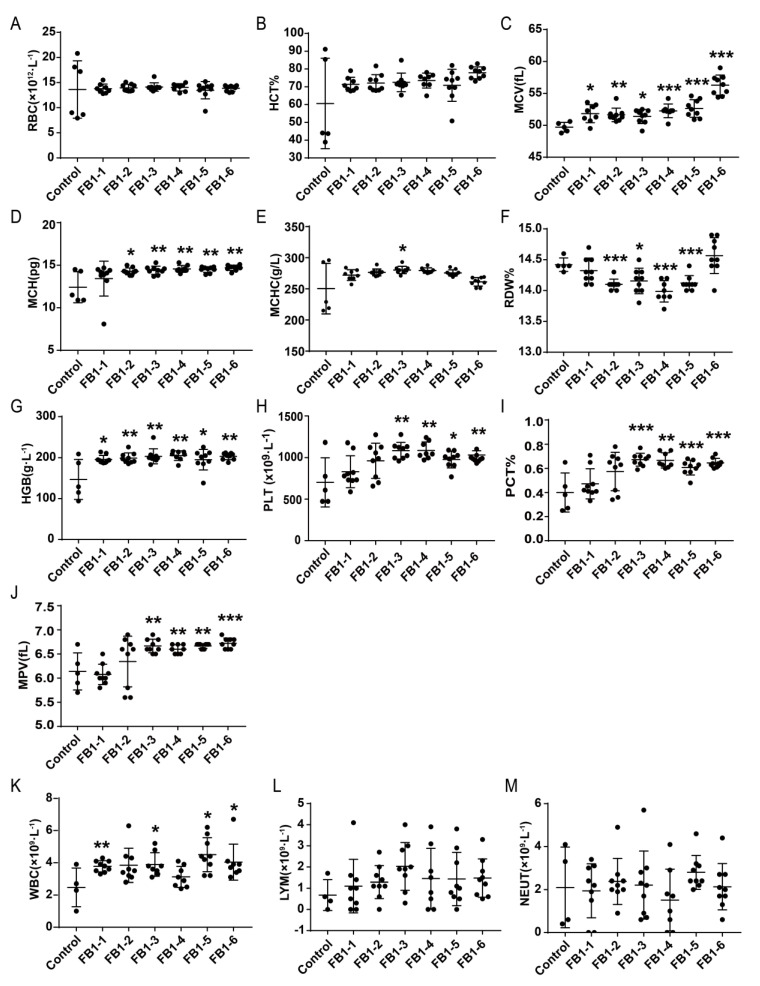
Effects of FB1 on hematology and related parameters in mice after 8 weeks of administration of FB1. (**A–G**): The changes of red blood cells (RBC) and related parameters. (**H–J**): The changes of PLT and related parameters. K to M: The changes of white blood cells (WBC) and related parameters. Values are shown as the mean ± SEM (*n* = 9); 2 weeks (*n* = 5), 4 weeks (*n* = 6–7), 6 weeks (*n* = 5), and 8 weeks (*n* = 9). FB1-1 indicates 0.018 mg/kg BW, FB1-2 indicates 0.054 mg/kg BW, FB1-3 indicates 0.162 mg/kg BW, FB1-4 indicates 0.486 mg/kg BW, FB1-5 indicates 1.458 mg/kg BW and FB1-6 indicates groups 4.374 mg/kg BW; * indicates *p* < 0.05, ** indicates *p* < 0.01, *** indicates *p* < 0.001, vs. the control group.

After 8 weeks of administration of FB1, the changes of red blood cells (RBC) and related parameters were shown in Figure 2A–G. Compared with the control group, the levels of mean corpuscular volume (MCV) and hemoglobin (HGB) in all FB1-treated groups were significantly increased (Figure 2C,G). Mean corpuscular hemoglobin (MCH) also presented a significant increase in the FB1-2 (0.054 mg/kg BW), FB1-3 (0.162 mg/kg BW), FB1-4 (0.486 mg/kg BW), FB1-5 (1.458 mg/kg BW) and FB1-6 groups (4.374 mg/kg BW) (Figure 2D). For red cell volume distribution width (RDW), its level was decreased in the FB1-2 (0.054 mg/kg BW), FB1-3 (0.162 mg/kg BW), FB1-4 (0.486 mg/kg BW) and FB1-5 (1.458 mg/kg BW) groups (Figure 2F). However, there was no significant difference in the levels of RBC, red blood cell specific volume (HCT) and mean corpuscular hemoglobin concentration (MCHC) (Figure 2A,B,E).

There were also some significant changes in the levels of PLT and related parameters after treatment with FB1 for 8 weeks. In the FB1-3 (0.162 mg/kg BW), FB1-4 (0.486 mg/kg BW), FB1-5 (1.458 mg/kg BW) and FB1-6 groups (4.374 mg/kg BW), platelet (PLT), plateletocrit (PCT) and mean platelet volume (MPV) were higher than those of the control group (Figure 2H–J).

The number of white blood cells (WBC) showed a dramatic increase in the FB1-1 (0.018 mg/kg BW), FB1-3 (0.162 mg/kg BW), FB1-5 (1.458 mg/kg BW) and FB1-6 (4.374 mg/kg BW) groups (Figure 2K), but the levels of lymphocytes (LYM) and neutrophils (NEUT) had no changes (Figure 2L,M).

### 2.3. Effects of FB1 on Blood Chemistry Parameters of Mice

**Figure 3:**
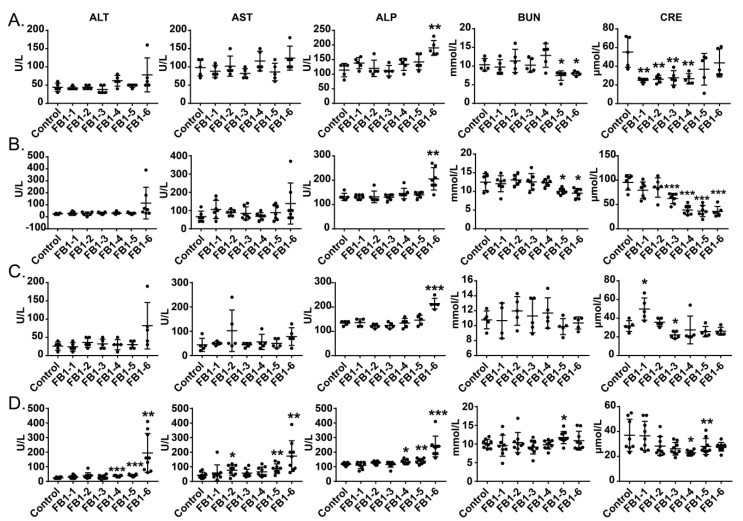
(**A**–**D**) Effects of FB1 on the blood chemistry parameters of mice exposed to different levels of FB1 for 2, 4, 6 and 8 weeks. Values are shown as the mean ± SEM; 2 weeks (*n* = 5), 4 weeks (*n* = 6–7), 6 weeks (*n* = 5), and 8 weeks (*n* = 9). FB1-1 indicates 0.018 mg/kg BW, FB1-2 indicates 0.054 mg/kg BW, FB1-3 indicates 0.162 mg/kg BW, FB1-4 indicates 0.486 mg/kg BW, FB1-5 indicates 1.458 mg/kg BW and FB1-6 indicates groups 4.374 mg/kg BW; * indicates *p* < 0.05, ** indicates *p* < 0.01, *** indicates *p* < 0.001, vs. the control group.

As shown in Figure 3, the serum AST, ALT and ALP activities showed dose-associated and exposure time-associated increases. All three enzymes reached peak values in the FB1-6 (4.374 mg/kg BW) group, especially when FB1 was given for 8 weeks. Even the value of AST in the FB1-2 (0.054 mg/kg BW) group after 8 weeks of FB1 treatment also presented a significant increase. The ALP level increased significantly at the FB1-6 dose after FB1 was administered to mice for only 2 weeks.

For blood urea nitrogen (BUN) and creatinine (CRE), during the first 4 weeks of exposure to FB1, the levels of BUN in the FB1-5 (1.458 mg/kg BW) and FB1-6 (4.374 mg/kg BW) groups were decreased significantly compared with those in the control group, whereas at the end of the experiment, BUN in the FB1-5 (1.458 mg/kg BW) group was obviously increased. The fluctuation of the CRE level during the whole experiment was obvious. CRE in mice showed a significantly decreased tendency except for the FB1-1 group after 2 weeks of exposure to FB1.

### 2.4. Effects of FB1 on Histopathologic Changes

#### 2.4.1. Liver

**Figure 4:**
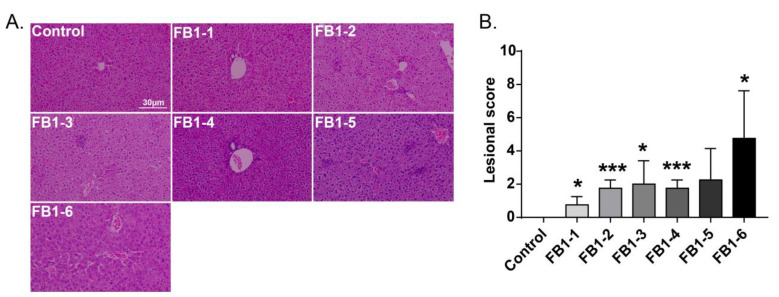
Histological changes in the liver in mice exposed to different doses of FB1 for 2 weeks. (**A**) Typical histological picture in each group (HE. 200×). (**B**) Lesional score after histological examination based on the occurrence and severity of lesions. Values are shown as the mean ± SEM (*n* = 5). FB1-1 indicates 0.018 mg/kg BW, FB1-2 indicates 0.054 mg/kg BW, FB1-3 indicates 0.162 mg/kg BW, FB1-4 indicates 0.486 mg/kg BW, FB1-5 indicates 1.458 mg/kg BW and FB1-6 indicates groups 4.374 mg/kg BW; * means *p* < 0.05, *** means *p* < 0.001, vs. control group.

**Figure 5:**
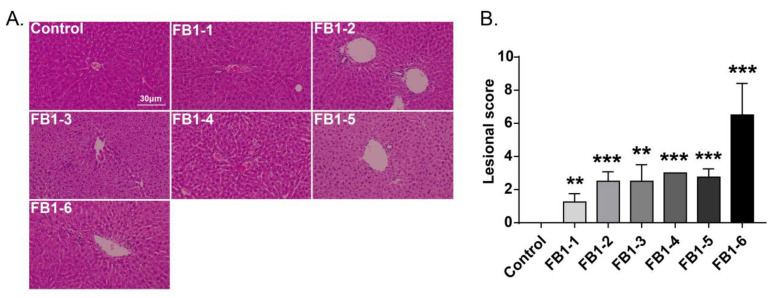
Histological changes in the liver in mice exposed to different doses of FB1 for 4 weeks. (**A**) Typical histological picture in each group (HE. 200×). (**B**) Lesional score after histological examination based on the occurrence and severity of lesions. Values are shown as the mean ± SEM (*n* = 6–7). FB1-1 indicates 0.018 mg/kg BW, FB1-2 indicates 0.054 mg/kg BW, FB1-3 indicates 0.162 mg/kg BW, FB1-4 indicates 0.486 mg/kg BW, FB1-5 indicates 1.458 mg/kg BW and FB1-6 indicates groups 4.374 mg/kg BW; * indicates *p* < 0.05, ** indicates *p* < 0.01, *** indicates *p* < 0.001, vs. the control group.

**Figure 6:**
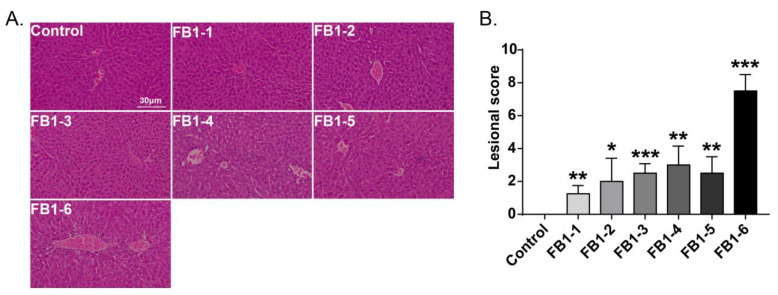
Histological changes in the liver in mice exposed to different doses of FB1 for 6 weeks. (**A**) Typical histological picture in each group (HE. 200×). (**B**) Lesional score after histological examination based on the occurrence and severity of lesions. Values are shown as the mean ± SEM (*n* = 5). FB1-1 indicates 0.018 mg/kg BW, FB1-2 indicates 0.054 mg/kg BW, FB1-3 indicates 0.162 mg/kg BW, FB1-4 indicates 0.486 mg/kg BW, FB1-5 indicates 1.458 mg/kg BW and FB1-6 indicates groups 4.374 mg/kg BW; * indicates *p* < 0.05, ** indicates *p* < 0.01, *** indicates *p* < 0.001, vs. control group.

**Figure 7:**
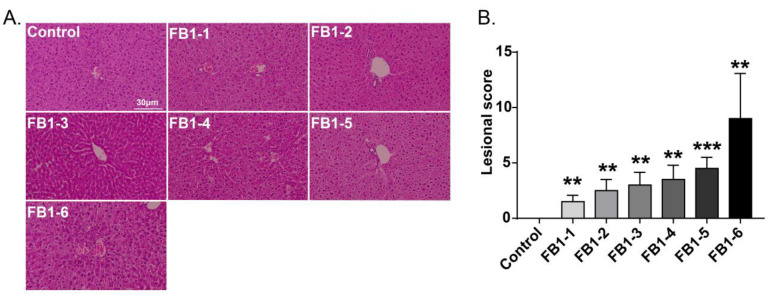
Histological changes in the liver in mice exposed to different doses of FB1 for 8 weeks. (**A**) Typical histological picture in each group (HE. 200×). (**B**) Lesional score after histological examination based on the occurrence and severity of lesions. Values are shown as the mean ± SEM (*n* = 9). FB1-1 indicates 0.018 mg/kg BW, FB1-2 indicates 0.054 mg/kg BW, FB1-3 indicates 0.162 mg/kg BW, FB1-4 indicates 0.486 mg/kg BW, FB1-5 indicates 1.458 mg/kg BW and FB1-6 indicates groups 4.374 mg/kg BW; * indicates *p* < 0.05, ** indicates *p* < 0.01, *** indicates *p* < 0.001, vs. control group.

Histological changes were found in the livers of mice in all FB1-treated groups. The main histological lesions observed in the liver were necroinflammation, including periportal necrosis and inflammation, intralobular necrosis and inflammation as well as portal inflammation. As shown in Figure 4, in the group of mice exposed to FB1-1 (0.018 mg/kg BW) and (0.054 mg/kg BW) for 2 weeks, infiltration of scattered inflammatory cells around the manifold area was already found. In the FB1-3 (0.162 mg/kg BW) group, a small number of hepatocytes started to exhibit necrosis. The effects were more severe in the FB1-5 (1.458 mg/kg BW) and FB1-6 (4.374 mg/kg BW) groups, as there were hepatocytes with vacuolar degeneration and detrital necrosis. From the results of lesional scores at 4, 6, and 8 weeks (Figure 4, Figure 5, Figure 6 and Figure 7), we also found that the lesional scores of all FB1-treated groups were significantly higher than those of the control group, even in the FB1-1 (0.018 mg/kg BW) group with the minimum dose of FB1. The lesional score increased in parallel with the increasing exposure time of FB1, and the highest score was found in the FB1-6 (4.374 mg/kg BW) group after 8 weeks of exposure to FB1.

#### 2.4.2. Kidneys

**Figure 8:**
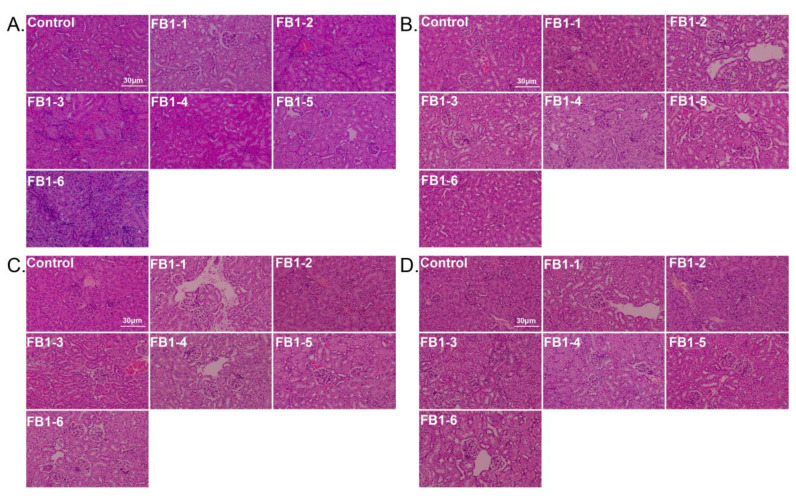
Histological changes in the kidneys in mice (HE. 200×). (**A**–**D**). Typical histological picture of mice suffering from different doses of FB1 for 2, 4, 6 and 8 weeks; 2 weeks (*n* = 5), 4 weeks (*n* = 6–7), 6 weeks (*n* = 5), and 8 weeks (*n* = 9). FB1-1 indicates 0.018 mg/kg BW, FB1-2 indicates 0.054 mg/kg BW, FB1-3 indicates 0.162 mg/kg BW, FB1-4 indicates 0.486 mg/kg BW, FB1-5 indicates 1.458 mg/kg BW and FB1-6 indicates groups 4.374 mg/kg BW.

In mice treated with different FB1 concentrations for different time periods, the main histological lesions observed in the kidneys were glomerular injury, renal tubular injury, and renal interstitial inflammation. Typical histological pictures are shown in Figure 8, and the corresponding lesional scores are shown in Table 1. Overall, vacuolar degeneration of renal tubular epithelial cells was ubiquitous in all FB1-treated groups and worsened over time. There was also tubular atrophy in some kidneys. For glomerular injury, slight mesangial cell hyperplasia first appeared in mice in the FB1-4 (0.486 mg/kg BW) group after exposure to FB1 for 4 weeks (Figure 8B), and the worst case occurred in FB1-6 (4.374 mg/kg BW) after the 8 week experiment (Figure 8D and Table 1).

## 3. Discussion

The liver and kidneys are the main target organs for the toxic effects of FB1 [21,22]. In this study, female BALB/c mice were exposed to different concentrations of FB1 by intragastric administration based on the fact that FB1 was more frequently ingested by the mouth. The liver and kidney toxicity of FB1 in mice was examined from two dimensions: dose and exposure time. Through the transformation of body surface area based on animal basal metabolic rate, the dose of 20 g mice is about 12.3 times that of 60 kg adults [23]. Therefore, the dose of FB1-1 (0.018 mg/kg BW) corresponds to the PMTDI (2 µg/kg BW) of FB1, FB2 and FB3, which is applied by the Joint FAO/WHO Expert Committee on Food Additives (JECFA). The results of this study suggested that the limit standard formulated by the FAO/WHO and the U.S. Food and Drug Administration (USFDA) is insufficient to protect humans and animals.

### 3.1. Body and Organ Weights

Since mice were exposed to FB1 for 4 weeks, there was a decreasing tendency in the body weights of mice, which reflected the cumulative alterations of FB1 treatment. Interestingly, exposure of FB1 for two weeks, high doses of FB1 (1.458 mg/kg BW and 4.374 mg/kg BW) actually increased the animal’s body weight. Previous studies have shown that low-dose aflatoxin is a hormesis for chickens [24]. Whether the short-term exposure of FB1 has the effect of increasing body weight may require further research. Changes in relative organ weights can better reflect the animal’s state and be reliable indicators of some physiological changes [25,26]. The results of this study showed that FB1 exposure had no significant effect on the relative heart weights of mice. However, the relative spleen weights increased first (2 weeks), and then the relative kidney weights increased (4 weeks), and finally the relative liver weights increased (8 weeks). The long-term effect of the minimum dose of FB1-1 (0.018 mg/kg BW) could also cause liver damage. The results of relative organ weights also suggested that FB1 might damage the immune system first and then damage the kidneys, finally leading to liver damage as the exposure time increased. This finding requires further research for confirmation.

### 3.2. Blood Chemistry

ALT and AST in serum are important indicators for reflecting liver function. ALT is an active enzyme mainly present in the liver, and AST has the highest content in the myocardium, followed by the liver. Both ALT and AST are released from damaged hepatocytes into the blood after hepatocellular injury or death [27]. ALP is an important indicator of cholestasis and hepatobiliary diseases in humans. Serum ALP levels can be elevated by cholestatic or infiltrative diseases of the liver and by diseases causing obstruction to the biliary system [27]. The results of this study showed that when FB1 was administered to mice for 8 consecutive weeks at the FB1-4 (0.486 mg/kg BW) dose, which was lower than the 4 mg/kg BW limit in corn flour and its products regulated by the USFDA, the ALT and ALP of mice was also significantly higher than that in the control group. The above results suggested that the dose of FB1 that is lower than the 4 mg/kg limit in corn flour and its products regulated by the USFDA has induced significant alterations in biochemical indicators.

### 3.3. Hematology

Routine blood tests are the most accessible and fundamental examination and have long been proposed as an essential assistant tool for disease diagnosis [28]. Changes in the blood system can predict the occurrence of some diseases. From our results, there were some significant hematological changes in female BALB/c mice after exposure to FB1. In particular, the MCV of mice in the low-dose FB1-1 (0.018 mg/kg BW) group changed significantly. The WBCs were estimated as an indicator of the immune response. Thus, Khawla Ezdini suggested that an inflammatory reaction to fight against mycotoxins impaired immunity [29]. In addition, MPV is a platelet volume index directly reflecting the platelet function state [30] and its elevation usually leads to thrombocytosis [29]. In our results, a significant increase in the number of WBCs existed in the low-dose FB1-1 (0.018 mg/kg BW) group and a significant increase in the number of PLTs and related parameters PCT and MPV in the low-dose FB1-3 (0.162 mg/kg BW) group. The results of hematology also suggested that the limit standard formulated by the FAO/WHO and USFDA is insufficient to protect humans and animals.

### 3.4. Histopathological Analysis

Exposure to FB1 resulted in injuries to the liver and kidneys, which could be directly reflected by histopathological analysis. After exposure to FB1 for 2 weeks, liver histological alterations were already observed at a dose of FB1-1 (0.018 mg/kg BW), which is lower than the limit standard formulated by the FAO/WHO. In terms of population, food consumption risk assessments should be performed in order to question the limit set by the FAO/WHO. In addition, the severity of lesions in the liver increased progressively, and dose-dependent severity was observed. The degree of lesions in the kidneys was not as severe as liver histological alterations, which was consistent with the finding that female mice were more sensitive to the hepatotoxic effects of FB1 than their male counterparts [31].

## 4. Conclusions

FB1 can cause significant hepatotoxicity, nephrotoxicity and hematological toxicity. Renal toxicity precedes hepatotoxicity, and the toxicity exhibits a certain dose dependence and exposure time dependence, especially histological alterations in the liver. The limit of the maximum tolerable daily intake of fumonisin in foods set by the FAO/WHO of 2 μg/kg body weight/day does not seem to have sufficient protection. FB1-1 (0.018 mg/kg BW), which is lower than this limit based on the dosage transformation of body surface area, can cause obvious deleterious influences on the liver and kidneys in female BALB/c mice, such as hepatocyte degeneration, necrosis and inflammation in the manifold and renal tubular damage in the kidneys. The FB1-4 dose (0.486 mg/kg BW) is slightly lower than the limit made by the USFDA for fumonisins in cornmeal and its products, and the FB1-1 (0.018 mg/kg BW), FB1-2 (0.054 mg/kg BW), and FB1-3 (0.162 mg/kg BW) doses are all significantly lower than this limit. These four doses of FB1 showed more significant toxic effects on mice. In short, the current regulatory limits for fumonisins are not sufficiently protective. Therefore, there would be more interest and importance in conducting further studies to determine the levels with the lack of observed adverse effects for fumonisins.

## 5. Materials and Methods

### 5.1. FB1 Solution Preparation

The FB1 solution was prepared by dissolving FB1 powder (Pribolab, Qingdao, China) in distilled water. First, we obtained a 0.4374 mg/mL FB1 solution and then diluted it with distilled water to reach concentrations of 0.1458 mg/mL, 0.0486 mg/mL, 0.0162 mg/mL, 0.0054 mg/mL and 0.0018 mg/mL.

### 5.2. Animal Trial

In this study, 181 female BALB/c mice (SPF grade, HFK Bioscience Co., Ltd. Beijing, China) with a body weight of 16–18 g and no specific pathogens (SPF) were used. The mice were kept in the Animal Experimental Center, Institute of Materia Medica, CAMS & PUMC with no restricted access to commercial feed and water. There were 4–5 mice kept in each cage. They were maintained in an environment at 20 ± 3 °C, 50.0 ± 10.0% humidity and a 12-h light/dark cycle. After acclimated for one week the mice were randomly divided into 7 groups according to body weight, which were labeled as the FB1-1 (0.018 mg/kg BW, 26 mice), FB1-2 (0.054 mg/kg BW, 26 mice), FB1-3 (0.162 mg/kg BW, 26 mice), FB1-4 (0.486 mg/kg BW, 26 mice), FB1-5 (1.458 mg/kg BW, 26 mice), FB1-6 (4.374 mg/kg BW, 26 mice), and the control group (25 mice). The doses of FB1-4 (0.486 mg/kg BW) and FB1-5 (1.458 mg/kg BW) crossed over the recommended levels of 4 mg/kg BW for total fumonisins (FB1 + FB2 + FB3) in whole or partially degermed dry milled corn products (e.g., flaking grits, corn grits, corn meal, corn flour with fat content of >2.25%, dry weight basis) by the USFDA. The control group was gavage with distilled water, and the other groups were gavage with corresponding FB1 solution. The feeding volume was 10 mL/kg per mouse each time and once a day. These similar treatments lasted for 2 (*n* = 5), 4 (*n* = 6–7), 6 (*n* = 5), and 8 (*n* = 9) weeks. On the last day of the 2, 4, 6, and 8 week periods, the mice were anesthetized by pentobarbital (80 mg/kg BW, given intraperitoneally (i.p.)). After collecting blood from the retro-orbital plexus, the animals were euthanasia by cervical dislocation and immediately dissected. The study was conducted according to the guidelines of the Declaration of Helsinki, and approved by The Animal Care & Welfare Committee, Institute of Materia Medica, CAMS & PUMC (protocol code 00003407. Approval Date: 30 October 2018).

### 5.3. Hematological Analysis

Exactly 20 μL of blood for hematological analysis were dispensed into test tubes containing anticoagulant. This was used for whole-blood analysis using an automatic blood analyzer following the instruction manual. The red blood cells (RBC), haemoglobin (HGB) red blood cell specific volume (HCT), mean corpuscular volume (MCV), mean corpuscular haemoglobin (MCH), mean corpuscular haemoglobin concentration (MCHC), RBC distribution width (RDW), white blood cells (WBC), lymphocyte (LYM), neutrophil (NEUT), platelets (PLT), plateletocrit (PCT), mean platelet volume (MPV) and platelet distribution width (PDW) were determined.

### 5.4. Blood Chemistry Parameters Analysis

Whole blood from each mouse was centrifuged at 4000 rpm for 15 min to prepare serum. The serum aspartate aminotransferase (AST) activity, alanine aminotransferase (ALT) activity, alkaline phosphatase (ALP) activity, creatinine (CRE), and urea nitrogen (BUN) were determined by a TBA-40FR Chemistry Analyzer (TOSHIBA, Tokyo, Japan) with commercial diagnostic kits (Biosino Bio-Technology and Science Inc., Beijing, China).

### 5.5. Histopathological Analysis

After gross examination of the organs of mice at necropsy, the liver and kidneys were fixed with 4% paraformaldehyde buffer, embedded in paraffin and blocked. The tissue blocks were sectioned to 4 μm and stained with hematoxylin-eosin for light microscopic analysis.

For the liver, histopathological analysis was performed according to the Knodell grading system, and the histological activity index (HAI) (range, 0–18) was used to grade the histological changes in tissues [32]. The overall Knodell score is the sum of scores for periportal ± bridging necrosis (0–10), intralobular degeneration and focal necrosis (0–4), portal inflammation (0–4), and fibrosis (0–4). The lesional score in our results is the sum of the first 3 components, which is used to reflect the necroinflammatory activity index. For the kidneys, the main pathological alterations were described and scored according to their extent and severity as follows: 0 indicated that a lesion was not present and 1–3 indicated that lesions were slight, moderate and severe.

### 5.6. Statistical Analysis

Statistical significance was determined following the test using either one-tailed *t*-test or one-way analysis of variance (ANOVA) with Tukey’s multiple comparisons to compare the means of multiple groups. Data are shown as the mean ± SEM and were considered statistically significant at *p* < 0.05. GraphPad Prism 7 (GraphPad Software Inc., LaJolla, CA, USA) was used for analysis and graphic building.

## Figures and Tables

**Table 1:** Lesional score of the kidneys in mice exposed to different levels of FB1 for 2, 4, 6 and 8 weeks.

**2 Weeks**	**Control**	**FB1-1**	**FB1-2**	**FB1-3**	**FB1-4**	**FB1-5**	**FB1-6**
Glomerulus	0.00 ± 0.00	0.00 ± 0.00	0.33 ± 0.58	0.00 ± 0.00	0.00 ± 0.00	0.00 ± 0.00	0.67 ± 0.58
Renal tubule	0.00 ± 0.00	0.33 ± 0.58	0.67 ± 0.58	1.00 ± 0.00	1.00 ± 1.00	0.67 ± 0.58	1.00 ± 0.00
Intestitial inflammation	0.00 ± 0.00	0.00 ± 0.00	1.33 ± 0.58 *	1.33 ± 0.58 *	0.67 ± 0.58	1.00 ± 0.00	2.00 ± 0.00
**4 Weeks**	**Control**	**FB1-1**	**FB1-2**	**FB1-3**	**FB1-4**	**FB1-5**	**FB1-6**
Glomerulus	0.00 ± 0.00	0.00 ± 0.00	0.00 ± 0.00	0.00 ± 0.00	0.33 ± 0.58	0.00 ± 0.00	0.00 ± 0.00
Renal tubule	0.00 ± 0.00	0.67 ± 0.58	1.00 ± 0.00	1.00 ± 0.00	0.67 ± 0.58	1.33 ± 0.58 *	0.67 ± 0.58
Intestitial inflammation	0.00 ± 0.00	0.00 ± 0.00	1.00 ± 0.00	0.67 ± 0.58	0.67 ± 0.58	0.33 ± 0.58	1.00 ± 0.00
**6 Weeks**	**Control**	**FB1-1**	**FB1-2**	**FB1-3**	**FB1-4**	**FB1-5**	**FB1-6**
Glomerulus	0.00 ± 0.00	0.00 ± 0.00	0.00 ± 0.00	0.67 ± 0.58	0.67 ± 0.58	0.67 ± 0.58	0.67 ± 0.58
Renal tubule	0.00 ± 0.00	0.67 ± 0.58	0.33 ± 0.58	1.00 ± 0.00	1.33 ± 0.58 *	0.67 ± 0.58	1.33 ± 0.58 *
Intestitial inflammation	0.00 ± 0.00	0.33 ± 0.58	1.00 ± 0.00	0.67 ± 0.58	0.00 ± 0.00	0.33 ± 0.58	0.67 ± 0.58
**8 Weeks**	**Control**	**FB1-1**	**FB1-2**	**FB1-3**	**FB1-4**	**FB1-5**	**FB1-6**
Glomerulus	0.00 ± 0.00	0.00 ± 0.00	0.67 ± 0.58	0.67 ± 0.58	1.00 ± 0.00	1.00 ± 0.00	1.00 ± 0.00
Renal tubule	0.00 ± 0.00	1.00 ± 0.00	0.67 ± 0.58	1.33 ± 0.58 *	1.00 ± 0.00	1.00 ± 0.00	1.67 ± 0.58 *
Intestitial inflammation	0.00 ± 0.00	0.00 ± 0.00	0.33 ± 0.58	0.67 ± 0.58	0.00 ± 0.00	0.33 ± 0.58	1.00 ± 0.00

Values are shown as the mean ± SEM; 2 weeks (*n* = 5), 4 weeks (*n* = 6–7), 6 weeks (*n* = 5), and 8 weeks (*n* = 9). FB1-1 indicates 0.018 mg/kg BW, FB1-2 indicates 0.054 mg/kg BW, FB1-3 indicates 0.162 mg/kg BW, FB1-4 indicates 0.486 mg/kg BW, FB1-5 indicates 1.458 mg/kg BW and FB1-6 indicates groups 4.374 mg/kg BW; Values are shown as the mean ± SEM; 2 weeks (*n* = 5), 4 weeks (*n* = 6–7), 6 weeks (*n* = 5), and 8 weeks (*n* = 9); * indicates *p* < 0.05 vs. control group.

## Data Availability

Data available on request due to restrictions e.g., privacy or ethical.
